# High-Throughput Non-destructive Phenotyping of Traits that Contribute to Salinity Tolerance in *Arabidopsis thaliana*

**DOI:** 10.3389/fpls.2016.01414

**Published:** 2016-09-28

**Authors:** Mariam Awlia, Arianna Nigro, Jiří Fajkus, Sandra M. Schmoeckel, Sónia Negrão, Diana Santelia, Martin Trtílek, Mark Tester, Magdalena M. Julkowska, Klára Panzarová

**Affiliations:** ^1^Division of Biological and Environmental Sciences and Engineering, King Abdullah University of Science and TechnologyThuwal, Saudi Arabia; ^2^Institute of Plant and Microbial Biology, University of ZurichZurich, Switzerland; ^3^PSI (Photon Systems Instruments)Drásov, Czech Republic

**Keywords:** high-throughput phenotyping, *Arabidopsis thaliana*, salt stress, salinity tolerance, shoot-ion independent tolerance, kinetic chlorophyll fluorescence imaging, color segmentation

## Abstract

Reproducible and efficient high-throughput phenotyping approaches, combined with advances in genome sequencing, are facilitating the discovery of genes affecting plant performance. Salinity tolerance is a desirable trait that can be achieved through breeding, where most have aimed at selecting for plants that perform effective ion exclusion from the shoots. To determine overall plant performance under salt stress, it is helpful to investigate several plant traits collectively in one experimental setup. Hence, we developed a quantitative phenotyping protocol using a high-throughput phenotyping system, with RGB and chlorophyll fluorescence (ChlF) imaging, which captures the growth, morphology, color and photosynthetic performance of *Arabidopsis thaliana* plants in response to salt stress. We optimized our salt treatment by controlling the soil-water content prior to introducing salt stress. We investigated these traits over time in two accessions in soil at 150, 100, or 50 mM NaCl to find that the plants subjected to 100 mM NaCl showed the most prominent responses in the absence of symptoms of severe stress. In these plants, salt stress induced significant changes in rosette area and morphology, but less prominent changes in rosette coloring and photosystem II efficiency. Clustering of ChlF traits with plant growth of nine accessions maintained at 100 mM NaCl revealed that in the early stage of salt stress, salinity tolerance correlated with non-photochemical quenching processes and during the later stage, plant performance correlated with quantum yield. This integrative approach allows the simultaneous analysis of several phenotypic traits. In combination with various genetic resources, the phenotyping protocol described here is expected to increase our understanding of plant performance and stress responses, ultimately identifying genes that improve plant performance in salt stress conditions.

## Introduction

Climate change and population growth place a twofold pressure on agricultural crop production. Crop yields need to be sustained and increased while grown in unfavorable environments (Godfray et al., 2010; Tester and Langridge, 2010; Tilman et al., 2011). To meet future food demands, breeding efforts are targeting more resource-efficient and stress-tolerant crops by combining large-scale plant phenotyping with genome sequencing in forward genetics studies. Phenotypic traits, including growth rate, size, shape, color, temperature and photosynthetic activity, are traditionally studied to evaluate plant performance under stress (Sirault et al., 2009; Zhang et al., 2012; Hairmansis et al., 2014; Chen et al., 2015; Ghanem et al., 2015). Plant breeding programs aimed at enhancing plant performance should investigate growth and photosynthetic activity in tandem because these processes are interdependent (Longenberger et al., 2009).

Advances in non-destructive image-based phenotyping technologies are enabling parallel studies of plant growth and photosynthetic performance over time (Munns et al., 2010; Dhondt et al., 2013; Hairmansis et al., 2014) using RGB and chlorophyll fluorescence (ChlF) imaging (Dhondt et al., 2013; Brown et al., 2014; Humplik et al., 2015). Traits related to plant growth, architecture and development have been quantified from digital color imaging, while leaf color is a simple, under-utilized trait that indicates plant health and leaf senescence (Berger et al., 2012). Kinetic ChlF imaging is a powerful tool for measuring plant photosynthetic capacity and provides valuable insights into the performance of photosynthetic apparatus (Oxborough, 2004; Baker, 2008). Light energy, captured by chlorophyll molecules, can undergo one of three fates: (1) be used to drive photosynthesis by photochemistry, (2) be dissipated as heat, or (3) be re-emitted as fluorescence. Because these three processes co-exist in close competition, the ChlF yield provides information on both the quantum efficiency of the plant’s photochemistry and on the amount of heat dissipated. Under conditions of stress, the photochemical yield decreases, which in turn, causes heat dissipation and ChlF emissions to increase (Maxwell and Johnson, 2000; Murchie and Lawson, 2013). Although, high-throughput phenotyping of photosynthetic performance has previously been employed to study plant response to cold (Jansen et al., 2009; Humplik et al., 2015) and drought (Bresson et al., 2015), few studies have established an integrative approach that simultaneously analyzes plant growth and photosystem II (PSII) efficiency (Humplik et al., 2015). Systems for kinetic ChlF imaging have not been widely integrated into high-throughput phenotyping platforms, in contrast to single-level steady-state ChlF imaging. The latter only reflects chlorophyll content and not PSII activity. This means that using single-level steady-state ChlF can only discriminate between healthy, senescing and stressed leaves by the amount of chlorophyll they degrade (Campbell et al., 2015).

Soil salinity is a key stress factor that affects agriculture on a global scale (Munns and Tester, 2008; Cabot et al., 2014; Roy et al., 2014). In saline soil, plants accumulate ions in their shoots, compromising plant growth with ion toxicity, for example by reducing the rate of photosynthesis (Munns and Tester, 2008). During the early phase of salt stress, before ions accumulate significantly in the shoots, the osmotic phase of salinity tolerance takes place, which is referred to as shoot ion-independent tolerance (SIIT; Roy et al., 2014). During this phase, growth reduction is caused by decreased leaf emergence and expansion (Fricke et al., 2006; Munns and Tester, 2008; Berger et al., 2012). Mechanisms underlying ion sensing, cell cycle, cell expansion, and stomatal conductance are likely underlying this response (Ma et al., 2006; Stephan and Schroeder, 2014). Thus, performing regular growth measurements from when salt stress is introduced to the plant across an extended period of time provides the opportunity to discriminate between early (osmotic) and late (ionic) growth-related responses to salt stress. During the early phase of salt stress, the capacity of photosynthetic machinery is reduced (James et al., 2006; Chaves et al., 2009). In later phases, excessive photonic energy causes photochemical inactivation (Muranaka et al., 2002) that reduces PSII stability (Stepien and Johnson, 2009), limits stomatal gas diffusion and causes changes in carbon assimilation rates (Chaves et al., 2009), ultimately resulting in decreased photosynthetic activity. Additional limits to photosynthesis may be caused by the accumulation of unused organic compounds from carbon assimilation (Munns and Tester, 2008). Early responses of plants to salinity have previously been quantified by measuring rosette area, color, temperature, and photosynthetic activity using steady-state ChlF (Rajendran et al., 2009; Sirault et al., 2009; Berger et al., 2012; Chen et al., 2015). Here, we quantify how traits related to plant morphology, color and photosynthetic activity respond to salinity in one experimental setup to establish significant correlations among individual phenotypes.

We developed a phenotyping method that monitors plant responses to salt stress by evaluating plant growth, color and photosynthetic traits using an automated, high-throughput system. We grew *Arabidopsis* plants in soil and maintained them at 40, 60, or 80% of the soil-water holding capacity to achieve approximately 150, 100, and 50 mM NaCl, respectively. We established 100 mM NaCl as the optimal condition for salt treatment. To characterize the early and late plant responses to salt stress, we investigated RGB, greenness and photosynthesis-related traits. Traits of ChlF were clustered with relative plant performance values into groups corresponding to early and late responses to salt stress. This work provides the means for screening natural diversity panels and mapping populations to identify candidate genes underlying plant development and stress tolerance.

## Materials and Methods

### Plant Material and Growing Conditions

**FIGURE 1:**
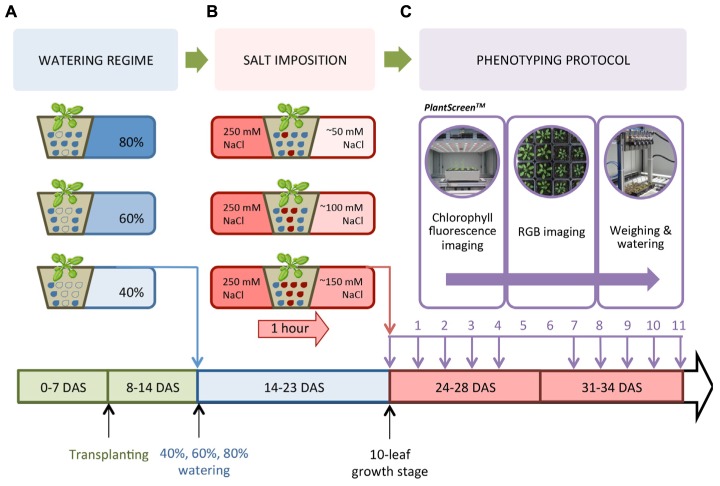
**Watering regime, salt stress treatment and phenotyping protocol.** **(A)** Col-0 and C24 were sown, watered to full soil-water saturation, stratified, then germinated under short day conditions. Similar-sized seedlings were transplanted into freshly sieved soil 7 days after stratification (DAS). At 14 DAS, watering was controlled to reach 80, 60, or 40% of the soil-water holding capacity. **(B)** Seedlings at the 10-leaf stage (24 DAS) were saturated for 1 h in a 250 mM NaCl solution to reach concentrations of 50, 100, and 150 mM NaCl, while control pots were saturated in dH_2_O. **(C)** The PlantScreen^TM^ Compact System performed chlorophyll fluorescence (ChlF) and RGB imaging, as well as automatically weighing and watering the plants. The lower panel depicts the timeline of the experiment.

Accessions of Arabidopsis Columbia-0 (Col-0) and C24 were used to establish the cultivation, salt treatment and phenotyping protocol. Thereafter, nine accessions of Arabidopsis [Col-0, C24, Canary Islands (Can), Coimbra (Co), Cape Verde Islands (Cvi), Landsberg *erecta* (Ler), Niederzenz (Nd), Rschew (Rsch) and Tenela (Te)] were used to optimize this protocol and investigate the natural variation of plants in response to salt stress (Hannah et al., 2006). Seeds were sown into pots (70 mm × 70 mm × 65 mm, Poppelman TEKU DE) containing 60 g of freshly sieved soil (Substrate 2, Klasmann-Deilmann GmbH, Germany) and watered to full soil-water holding capacity. Seeds were stratified for 3 days at 4°C in the dark. All plants were grown in a climate controlled growth chamber (FS_WI, PSI, Czech Republic) with cool-white LED and far-red LED lighting. The protocol was setup with Col-0 and C24 grown in a 10 h/14 h 21°C/15°C light/dark cycle at a relative humidity of 60% and a photon irradiance of 250 μmol m^-2^ s^-1^. The protocol was optimized for the nine accessions using a 12 h/12 h 22°C/20°C light/dark cycle with a relative humidity of 55% and an irradiance of 150 μmol m^-2^ s^-1^. Seven days after stratification (DAS), seedlings of similar size were transplanted into soil that had been watered 1 day in advance to full soil-water holding capacity. Plants were cultivated in the growth chamber until most plants were at the 10-leaf stage (24 DAS for plants in the 10 h/14 h light/dark cycle and 21 DAS in the 12 h/12 h light/dark cycle). The growth timeline for Col-0 and C24 plants illustrates the implementation of the three watering regimes, the salt treatment and the phenotyping protocol (**Figure 1**).

### Watering and Salt Treatment

Similar to Junker et al. (2015), we determined the soil water-holding capacity by filling 10 pots with 60 g of sieved soil and drying them for 3 days at 80°C to completely desiccate the soil. Soil was then saturated with water and left to drain for 1 day before weighing. Based on the soil-water content at 100% (130 g), 40, 60, and 80% of the soil-water holding capacity were found to weigh 52, 77, and 103 g, respectively. At 14 DAS, Col-0 and C24 seedlings were placed randomly in trays (5 × 4 pots per tray) and into the PlantScreen^TM^ Compact System (PSI, Czech Republic) and automatically weighed and watered every other day to reach and maintain the reference weight corresponding to the desired soil-water contents (**Figure 1A**). Once at the 10-leaf stage at 24 DAS, nine replicates per accession were placed in 250 mM NaCl or dH_2_O for 1 h to ensure full saturation of the soil. Pots were left to drain for 10 min before being placed in the phenotyping system. Effective NaCl concentration in the soil was estimated as 150, 100, and 50 mM NaCl in plants watered to 40, 60, and 80% soil-water holding capacity, respectively, representing conditions of severe, moderate, and mild salt stress (**Figure 1B**). For 11 days, with the exception of days 5 and 6 (**Figure 1C**), plants were transferred manually from the growth chamber to PlantScreen^TM^ for image acquisition and then returned to the same positions inside the growth chamber. On the final day of imaging, the water content of the soil was found to be approximately 60–70%, indicating that plants had adequate soil moisture during the phenotyping period.

After analyzing the effects of the three watering regimes, 60% soil-water content and 100 mM NaCl were established as effective conditions for investigating early responses to salt stress. We then optimized the protocol, in terms of growth conditions and ChlF imaging, using nine accessions of Arabidopsis. At 18 and 20 DAS pots were watered to the target soil-water content. Once at the 10-leaf stage, at 21 DAS, eight replicates per accession were placed in a 250 mM NaCl solution or dH_2_O for 1 h to ensure saturation of the soil. Plants were imaged for 7 days with no additional watering. The final soil-water content was approximately 70–80%.

### High-Throughput Phenotyping

Control and salt-treated plants were automatically phenotyped for RGB and kinetic ChlF traits using PlantScreen^TM^ (**Supplementary Figures S1** and **S2**) from 1 h after introducing salt stress. The phenotyping was conducted for 11 days to develop the protocol and 7 days to investigate natural variation among the nine accessions. Trays were transported within PlantScreen^TM^ on conveyor belts between the light-isolated imaging cabinets, weighing and watering station and the dark/light acclimation chamber. A single round of measuring consisted of an initial 15 min dark-adaptation period inside the acclimation chamber, followed by ChlF and RGB imaging, weighing and watering. Pixel count, color and fluorescence intensity were evaluated from the images. A total time of 1 h and 40 min was required to measure 10 trays (200 plants). The PlantScreen^TM^ Analyzer software (PSI, Czech Republic) was used to automatically process the raw data.

#### RGB Imaging and Processing

Trays were loaded into the imaging cabinet of the PlantScreen^TM^ platform with three RGB cameras (one top and two side views) mounted on robotic arms, each supplemented with an LED-based light source to ensure homogenous illumination of the imaged object. To assess plant growth and morphological traits, RGB images (resolution 2560 × 1920 pixels) of 5 × 4 plants per tray were captured using the GigE uEye UI-5580SE-C/M 5 Mpx Camera (IDS, Germany) from the top view only. Light conditions, plant position and camera settings were fixed throughout the experiments. The PlantScreen^TM^ software required three steps to extract features from the RGB images. (1) Basic processing applied in real-time involving correction for barrel (fisheye) distortion, tray detection, cropping of individual pots, background subtraction to remove non-plant pixels from the images, filtration and artifact removal to produce binary (black and white) and RGB representations of each plant [binary images represent the plant’s surface (white) and background (black)]; (2) morphologic analysis, requiring separation of the background from the plant shoot tissue allowing the pixel number per plant and rosette area to be counted; and (3) analysis of greenness using background-subtracted RGB images to evaluate the color. For this step, the images were color-segmented to represent and evaluate rosette coloring (**Supplementary Figure S1**). The morphometric parameters area, perimeter, roundness, compactness, rotational mass symmetry, eccentricity and slenderness of leaves were computed from the RGB image processing and have been listed and defined in **Supplementary Table S1**.

#### Plant Growth-Related Parameters

**FIGURE 2:**
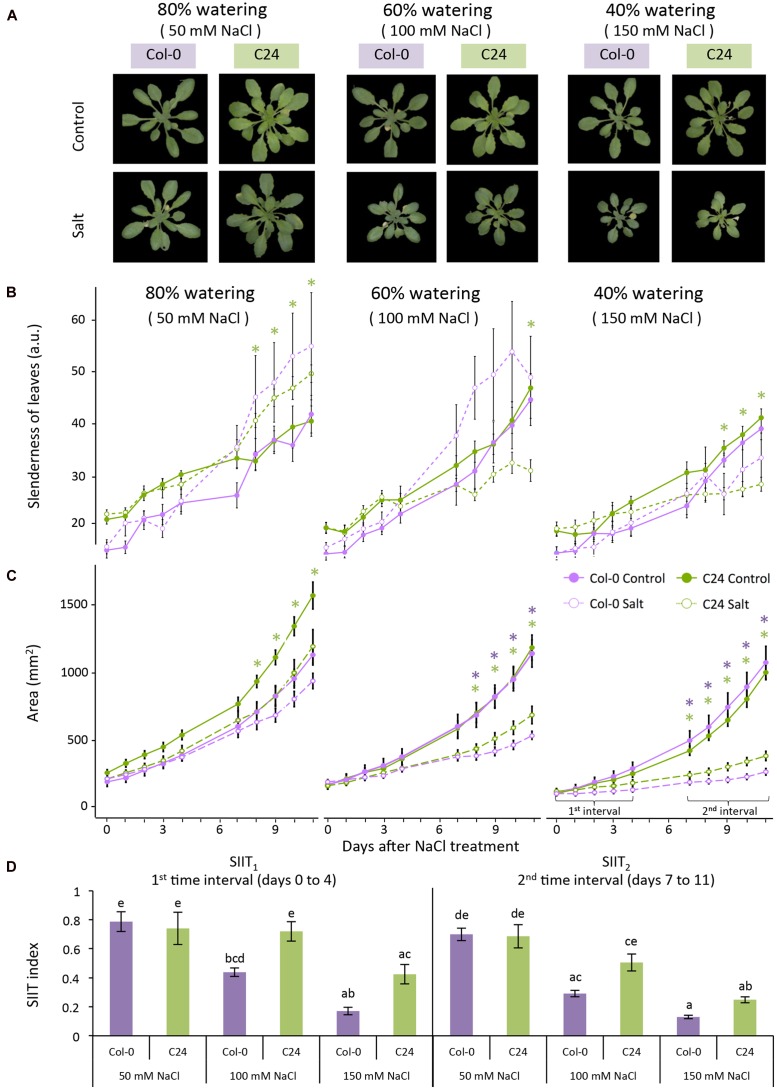
**Col-0 and C24 growth-related responses to salt stress.** **(A)** RGB images of control (upper panel) and 11 days salt-stressed (lower panel) plants maintained at 80, 60, and 40% of the soil water-holding capacity, which correspond to approximately 50, 100, and 150 mM NaCl, respectively. **(B)** Leaf slenderness and **(C)** projected rosette area over time for Col-0 (purple lines) and C24 (green lines) in control (solid lines) and salt-stressed (dashed lines) conditions. Values represent the averages of nine replicates per accession and treatment. Error bars represent standard error. The significant differences between control and salt treatment per accession are indicated with ^∗^ and ^∗∗^ for *p*-values below 0.05 and 0.01, respectively. **(D)** The shoot ion-independent tolerance (SIIT) index was calculated as the ratio of growth rates in salt-stressed conditions to average growth rates observed in control conditions per accession and treatment over two time intervals. Values represent the average of nine biological replicates per accession and treatment. Error bars represent standard error. Different letters are used to indicate the significant differences between the accessions and conditions as tested with one-way ANOVA with *post hoc* Tukey’s test (*p <* 0.05).

To evaluate the effect of salt stress on early and late plant growth rates (GR), we examined the increase in projected rosette area over time by fitting a linear function to two time intervals. The regression coefficient of the fitted function was determined and used as a trait in the statistical analysis. Relative effects of salt stress were calculated by dividing the estimated growth rates (GR) in salt conditions by the average in control conditions (GR _(salt)_/GR _(control)_). The calculation was performed for each accession over two time intervals (0–4 days and then 7–11 days when developing the protocol, and 0–3 days and then 4–7 days when examining natural variation among accessions; **Figures 2D** and **5C**). This ratio has been termed the shoot ion-independent tolerance (SIIT) index, which is used as a measure of plant salinity tolerance (Roy et al., 2014). The effects of salt stress were determined by performing an analysis of variance (ANOVA) per accession and treatment with Tukey’s *post hoc* test of significance for each RGB trait (*p*-value < 0.05).

#### Color Segmentation and Evaluation of Greenness

**FIGURE 3:**
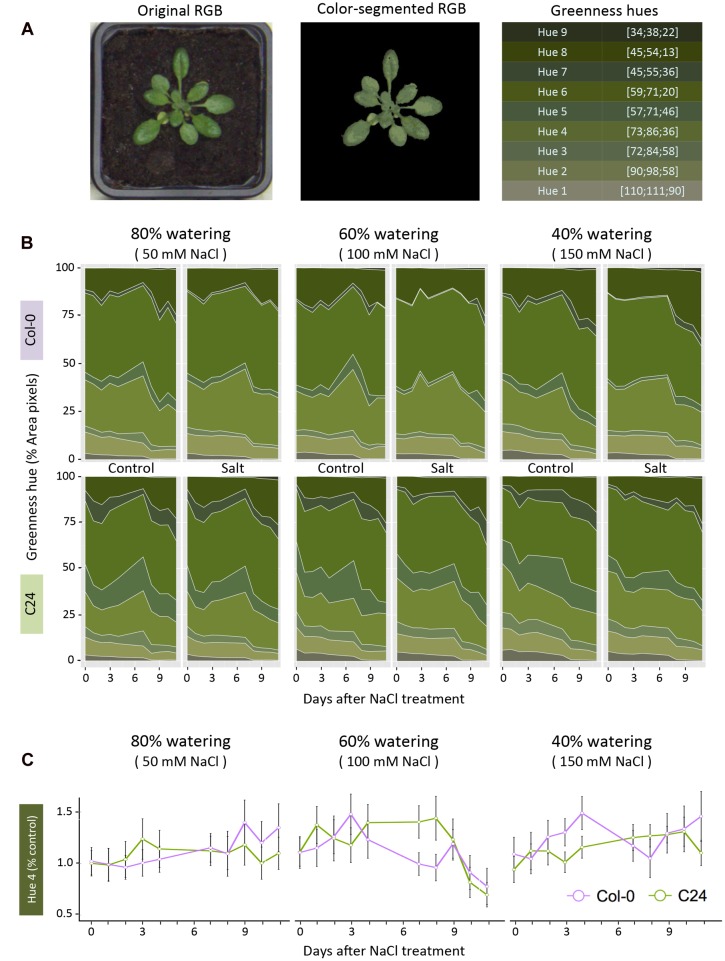
**Quantification of change in rosette color in response to salt stress.** **(A)** RGB images were color-segmented into nine green hues, which are summarized with their RGB coordinates. **(B)** Dynamic relative changes in greenness hue abundance are presented across the phenotyping period, with 50 mM NaCl (left panel), 100 mM NaCl (middle panel) and 150 mM NaCl (right panel), respectively. The hues ordered in the right panel of A are presented as a portion of the rosette area (pixel counts) in 80, 60, and 40% watering with 50, 100, and 150 mM NaCl, respectively. Values shown represent the averages calculated over nine biological replicates per accession and treatment. **(C)** Salt-induced changes in hue 4, calculated as relative to control conditions, for Col-0 (purple) and C24 (green). Values represent the averages of nine replicates per accession and treatment. Error bars represent standard error. The significant differences between control and salt-stressed treatments per accession are indicated with ^∗^ and ^∗∗^ for *p*-values below 0.05 and 0.01, respectively, as calculated with one-way ANOVA with *post hoc* Tukey’s test.

Using color segmentation, we analyzed the change in rosette coloring. We calibrated the analysis by using RGB images from both control and salt-stressed conditions and from the start, middle and end of the phenotyping period to obtain an unbiased color scale (**Supplementary Figure S1**). Values in the RGB channels, from each pixel corresponding to the plant’s surface area, were extracted to serve as a dataset for k-means clustering. Nine clusters were sufficient for optimal color differentiation and all input pixels were partitioned according to their Euclidean distance in the RGB color space. The RGB coordinates of cluster centroids were used as base hues to evaluate greenness. Original pixel color was approximated from the nearest cluster centroid, yielding color-segmented images. To calculate the relative hue abundance independent of the rosette area, pixel counts of individual hue values were divided by the rosette area of the same plant on the same day (**Figure 3**). The effect of salt stress was determined by performing an ANOVA per accession and treatment with Tukey’s *post hoc* test of significance for each greenness hue (*p-*value < 0.05).

#### Kinetic Chlorophyll Fluorescence Imaging and Processing

To assess the effect of salt stress on photosynthetic performance, ChlF measurements were acquired using an enhanced version of the FluorCam FC-800MF pulse amplitude modulated (PAM) chlorophyll fluorometer (PSI, Czech Republic). The ChlF imaging station was mounted on a robotic arm with an LED light panel and a high-speed charge-coupled device camera (pixel resolution of 720 × 560, frame rate 50 fps and 12-bit depth) positioned in the middle of the light panel (**Supplementary Figure S2**). The LED panel was equipped with 3 × 64 orange-red (618 nm) and 64 cool-white LEDs (6500 K), distributed equally over 75 × 75 cm. This resulted in a ±5% maximum deviation from the mean across the imaged area of 35 × 35 cm. Modulated light of known wavelength was applied to detect the ChlF signal. Three types of light sources were used: (1) PAM short-duration measuring flashes (33 μs) at 618 nm, (2) orange-red (618 nm) and cool-white (6500 K) actinic lights with maximum irradiance 440 μmol m^-2^ s^-1^ and (3) saturating cool-white light with maximum irradiance 3000 μmol m^-2^ s^-1^.

Plant trays were automatically loaded into the light-isolated imaging cabinet of PlantScreen^TM^ with a top-mounted LED light panel. After the 15 min dark-adaptation period, when PSII reaction centers open, the trays were automatically transported to the ChlF imaging cabinet. A 5 s flash of light was applied to measure the minimum level of fluorescence in the dark-adapted state (F_o_), followed by a saturation pulse of 800 ms (with an irradiance of 1200 μmol m^-2^ s^-1^) used to determine the maximum fluorescence in the dark-adapted state (F_m_). Plants were relaxed in the dark for 17 s and then subjected to 70 s of cool-white actinic lights to drive photosynthesis and measure the peak rise in fluorescence (F_p_). These conditions were used in both ChlF imaging techniques using quenching kinetics and light curve protocol.

For quenching kinetics protocol, additional saturation pulses were applied at 8, 18, 28, 48, 68 s during actinic illumination, corresponding to L1, L2, L3, L4, and Lss states at a constant photon irradiance of 210 μmol m^-2^ s^-1^ (**Supplementary Figure S3**). These ChlF signals were used to acquire the maximum fluorescence in the light-adapted state (F_m_′), and the level of ChlF measured just before the saturation pulse was considered the steady-state fluorescence in the light-adapted state (F_t_). Further responses to dark-relaxation were measured by switching the actinic light off for 100 s and applying saturating pulses at 30, 60, and 90 s, corresponding to D1, D2, D3 states (**Supplementary Figure S2**). The PlantScreen^TM^ Analyzer software performed the automated ChlF feature extraction by mask application, background subtraction and parameter calculation based on the fluorescence levels of F_o_, F_m_, F_p_, F_t_ and F_m_′, which were estimated by integrating pixel-by-pixel values across the entire rosette (**Supplementary Figure S2**; **Supplementary Table S2**). Minimum fluorescence in the light-adapted stated (F_o_′) was calculated according to Oxborough and Baker (1997).

For the examination of natural variation in the nine Arabidopsis accessions we optimized ChlF imaging by quantifying the rate of photosynthesis at different photon irradiances using the light curve protocol (Henley, 1993; Rascher et al., 2000) which was proven to provide detailed information on ChlF under stress (Brestic and Zivcak, 2013). A 5 s flash of light was applied to measure the minimum fluorescence, followed by a saturation pulse of 800 ms (with an irradiance of 1200 μmol m^-2^ s^-1^) to determine the maximum fluorescence in the dark-adapted state. Next, 60 s intervals of cool-white actinic light at 95, 210, 320, 440 μmol m^-2^ s^-1^ corresponding to L1, L2, L3, and L4, respectively, were applied. A saturation pulse was applied at the end of the period of actinic light to acquire the maximal fluorescence in the light-adapted state (**Supplementary Figure S6A**). The ChlF signal measured just before the saturation pulse was taken as the steady-state fluorescence value in the light-adapted state. The ChlF parameters were extracted and processed as described above for data collected from day 0 to day 7 of salt treatment (**Figure 6**).

#### Statistical Analysis on Chlorophyll Fluorescence-Related Responses to Salt Stress

**FIGURE 4:**
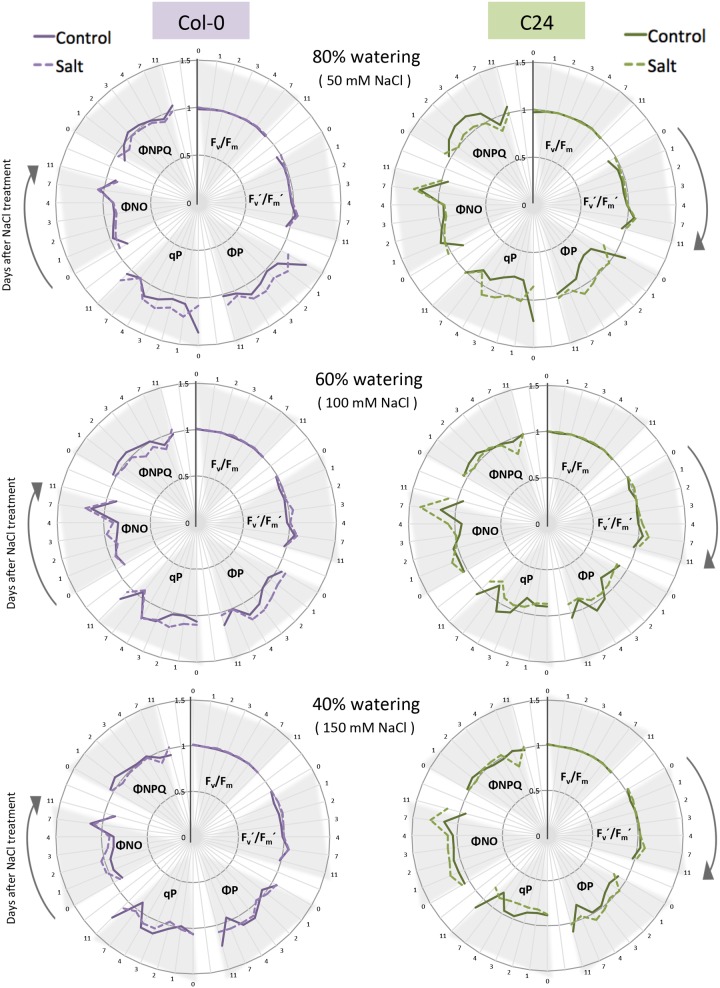
**The effect of watering regime and salt treatment on ChlF parameters.** Maximum quantum yield of PSII photochemistry for the dark-adapted (F_v_/F_m_) and light-adapted (F_v_′/F_m_′) states, the quantum yield of PSII photochemistry for the light-adapted state (ΦP), the photochemical quenching coefficient that estimates the fraction of open PSII reaction centers (qP), the quantum yield of constitutive non-regulatory non-photochemical dissipation processes (ΦNO) and the quantum yield of regulatory non-photochemical quenching (ΦNPQ) were measured using the quenching protocol for Col-0 (purple) and C24 (green) plants in control (solid line) and salt-stressed (dashed line) conditions. Time in days is indicated on the outer rim of the graph. Individual ChlF traits are presented in clusters indicated by gray triangles. Values represent the average of nine biological replicates per accession and treatment divided by the overall average per trait. Values were measured for plants grown in 80, 60, and 40% soil-water contents in control or salt-stressed conditions.

An ANOVA with Tukey’s *post hoc* test of significance (*p*-value < 0.05) was used to evaluate the differences in ChlF between control and salt-stressed plants. Trait values specific to accession, day and condition were divided by the overall average per trait to analyze the fluctuations in the ChlF traits, which were due to both plant development and salt treatment (**Figures 4** and **6**). Principal component analysis (PCA) was performed on 20 ChlF traits collected from Col-0 and C24 under the different adapted states and saturating pulses (L1 to L4, Lss and D1 to D3) to reduce data dimensionality (**Supplementary Figure S5**; **Supplementary Table S3**). Eight ChlF traits (F_v_/F_m_, F_v_′/F_m_′, ΦP, qP, ΦNO, ΦNPQ, qN and NPQ; Lazar, 2015) measured on day 7 at 440 μmol m^-2^ s^-1^ were clustered with SIIT_1_ and SIIT_2_ (GR_salt_/GR_control_ for each time interval) using the Ward linkage method. This was performed to study the relationships between the ChlF traits and relative changes in growth rate under salt stress among the nine accessions (**Figure 7**). Normalization was done by dividing the relative trait values by the overall average per trait. Mann–Whitney *U*-test with a continuity correction were performed on the ChlF parameters captured by the light curve protocol, and *p*-values were calculated using treatment as a grouping variable per accession for each day of the phenotyping period (**Supplementary Table S4**).

## Results

### Salt Stress Affected Growth and Morphology-Related Traits Over Time

To define the most suitable screening conditions to study early responses to salt stress, we first aimed to optimize the salt treatment (**Figure 1**). Three watering regimes were used to control the soil-water content (40, 60, and 80%; **Figure 1A**). The salt solution was diluted according to the watering regime to the final concentrations of 150, 100, or 50 mM NaCl in the soil corresponding to severe, moderate and mild salt stress, respectively (**Figure 1B**) and plants were phenotyped using RGB and ChlF imaging (**Figure 1C**). We then analyzed the phenotypes of Col-0 and C24 plants to discern the conditions most suitable for screening early salt-induced changes without greatly compromising plant health (**Figure 2**).

Investigation of rosette morphology revealed that Col-0 developed more slender leaves than C24 under control conditions (**Figure 2B**; **Supplementary Table S1**), but under severe salt stress, differences were less pronounced. Changes in roundness and compactness of Col-0 and C24 leaves were apparent after 2–3 days of salt treatment, whereas changes in rotational mass symmetry, eccentricity and slenderness of Col-0 leaves were recorded after 1 day of salt treatment (**Supplementary Table S1**; **Figure 2C**). C24 plants showed significant decreases in rosette area with salt treatment at day 8 in mild and moderate stress conditions and at day 7 in severe stress conditions. Mild stress levels did not cause a significant decrease in rosette area in Col-0 plants but a marked decrease was observable at day 8 with moderate and at day 7 with severe salt stress (**Figure 2C**).

Growth rates were estimated in each accession and condition by splitting the growth period into two intervals, 0–4 days and from 7 to 11 days, and fitting two linear functions to the increase in rosette area over time (**Figure 2C**). Growth rates of salt-treated plants were smaller in both intervals than those of control plants. This reduction was more pronounced in the second interval (**Supplementary Table S1**), allowing the discrimination between early and late responses to salt stress. The ratio of GR_salt_ to GR_control_ was used to describe the SIIT index and was calculated to assess salinity tolerance in the early (SIIT_1_) and late (SIIT_2_) phases of salt stress (**Figure 2D**). There was a clear decrease in SIIT values in both Col-0 and C24 plants with increasing salt stress levels. Both SIIT_1_ and SIIT_2_ of C24 were higher than those of Col-0 under moderate and severe salt stress conditions in both intervals, indicating that C24 has higher salinity tolerance. Differences between control and salt-stressed plants and between Col-0 and C24 were most pronounced under moderate and severe conditions of salt stress. In addition, control plants grown in the 40% soil-water content were much smaller than control plants grown in other watering regimes (**Figure 2C**), suggesting that these plants were likely to be suffering from drought stress. Mild salt stress had no effect on the growth of Col-0 and only a slight effect was observed later in C24 plants (**Figure 2B**). We established that plant growth and performance was best assessed using a 60% watering regime, which resulted in moderate salt stress of 100 mM NaCl.

### Color Segmentation of RGB Images Illustrates Changes in Rosette Greenness Over Time

We examined the features of pixel color in the RGB images to identify changes in rosette greenness under salt stress. Color information was extracted from pixels corresponding to the imaged rosettes. RGB images were color-segmented into nine green hues and analyzed for their relative abundance as a percentage of rosette area (**Figure 3A**). This strategy enabled the number of pixels representing each hue to be normalized for rosette area and compared between accessions and treatments (**Figure 3B**). Hues 1, 2, 5, and 8 changed over time without marked differences between treatments, while hues 3, 4, 6, 7, and 9 differed from control conditions after only 1 day of exposure to salt stress (**Figure 3B**). We calculated the ratio for each hue, between control and salt-treated plants, to observe the salt-induced changes among between the accessions and treatments. We presented the results for hue 4, showcasing the differences between Col-0 and C24 throughout the phenotyping period (**Figure 3C**).

### Chlorophyll Fluorescence Imaging Captures the Early and Late Changes in Photosynthetic Performance in Response to Salt Stress

To further explore the photosynthetic performance of control and salt-treated plants, we used ChlF parameters measured by the PAM method and quenching kinetics. From the measured fluorescence transient states, the basic ChlF parameters were derived (i.e., F_o_, F_m_, F_m_′, F_t_, F_v_, and F_p_), which were used to calculate the quenching coefficients (i.e., qP, NPQ, PQ, and qN) and other parameters characterizing plant photosynthetic performance (i.e., F_o_′, F_v_/F_m_, ΦP, F_v_′/F_m_′, ΦNO, ΦNPQ and Rfd; these parameters are summarized in **Supplementary Table S2**). The quenching kinetics protocol allowed to detect the shifts in the ChlF curves of Col-0 and C24 salt-stressed plants as early as 1 h after moderate or severe stress (**Supplementary Figure S3**).

We then selected six ChlF parameters that reflect the photosynthetic function of PSII (Lazar, 2015): the maximum quantum yield of PSII photochemistry in the dark-adapted (F_v_/F_m_), and the light-adapted (F_v_′/F_m_′) states, the coefficient of photochemical quenching that estimates the fraction of open PSII reaction centers (qP), the actual quantum yield of PSII photochemistry in the light-adapted state, the proportion of light absorbed by the chlorophyll associated with PSII that is used in photochemistry (ΦP), the quantum yield of constitutive non-light-induced dissipation consisting of ChlF emission and heat dissipation (ΦNO) and the quantum yield of regulatory light-induced heat dissipation (ΦNPQ) for days 0, 1, 2, 3, 4, 7, and 11 of the phenotyping period (**Figure 4**). The traits related to maximum quantum yield (F_v_/F_m_ and F_v_′/F_m_′) were not significantly different between control and salt-treated plants (**Figure 4**; **Supplementary Figure S4**; **Supplementary Table S2**), suggesting that PSII was not damaged during the course of the experiment. The other four ChlF parameters (qP, ΦP, ΦNO, and ΦNPQ) varied with time in both control and salt-stressed conditions (**Figure 4**).

Change in nine ChlF parameters, with respect to control conditions, was used to indicate the effect of salt stress on quenching processes and PSII efficiency (**Supplementary Table S2**; **Supplementary Figure S4**): traits measured in the light-adapted state were most affected (L1, L2, L3, L3, and Lss), while traits measured in the dark-adapted state (D1, D2, and D3) did not vary between treatments or accessions, except in the case of NPQ, qN, RFD, and ΦNPQ under severe stress (**Supplementary Figure S4**). F_v_′/F_m_′ was unchanged in all salt treatments and in both accessions and ΦNO displayed only slight changes indicating that those parameters were robust in response to salt stress. With this protocol we were able to detect rapid changes in ΦP, qP, PQ, NPQ, qN, RFD, and ΦNPQ in C24, but not in Col-0, after only 1 day of salt treatment (**Supplementary Figure S4**). To explore the ChlF parameters even further, we used PCA to classify the observed trends (**Supplementary Figure S5**). PCA performed on the 20 ChlF traits, under the eight different adapted states and saturating pulses (L1 to L4, Lss and D1 to D3), showed that the five PCs explained 85% of the variation (**Supplementary Figure S5**). PC1 described accession-specific trends and PC2 contained traits relevant to treatment with salt (**Supplementary Table S3**).

### Natural Variation of Growth-Related Traits was Quantified in Response to Salt Stress

**FIGURE 5:**
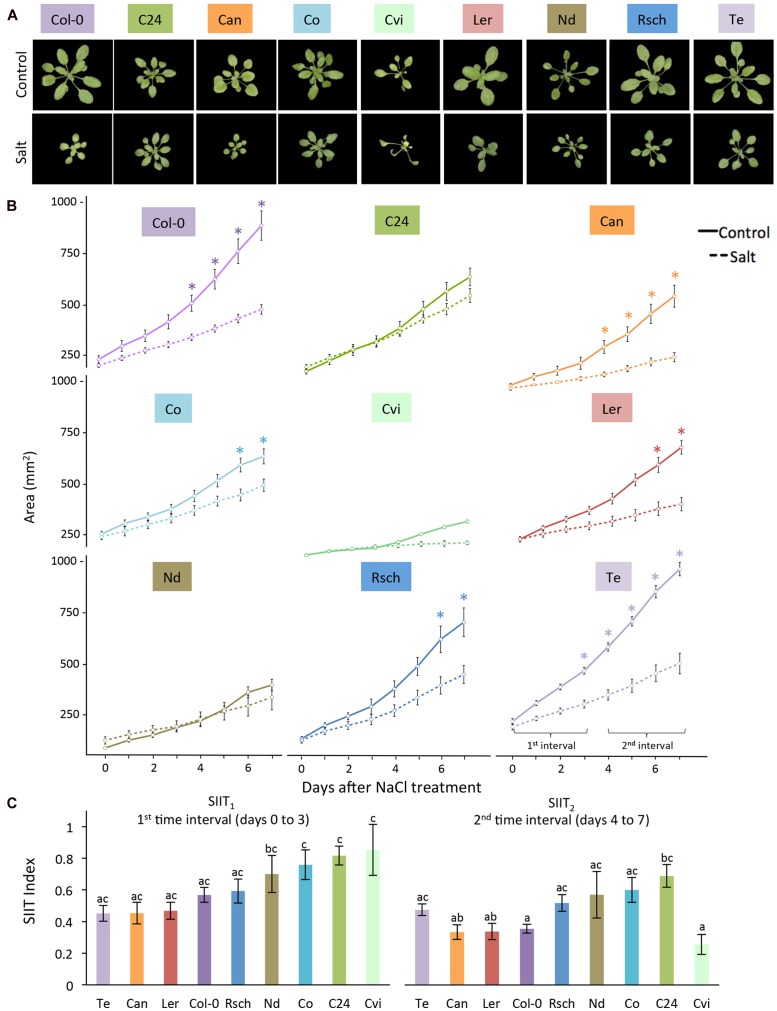
**Natural variation in growth-related responses of nine Arabidopsis accessions under salt stress.** **(A)** RGB images of control (upper panel) and 7 days salt-stressed (lower panel) plants. **(B)** Projected rosette area over time for plants grown in control (solid lines) and salt-stressed (dashed lines) conditions. Values represent the average of eight biological replicates per accession and treatment. Error bars represent standard error. Significant differences between control and salt stress treatment are indicated with ^∗^ and ^∗∗^ for *p*-values below 0.05 and 0.01, respectively. **(C)** Shoot ion-independent tolerance (SIIT_1_ and SIIT_2_) values were calculated from averages of eight replicates per accession and treatment. Error bars represent standard error. Different letters indicate significant differences between accessions as tested with one-way ANOVA with *post hoc* Tukey’s test (*p* < 0.05).

Because moderate salt stress elicited significant changes between control and salt-treated plants without symptoms of severe stress, it was used to investigate natural variation among the nine accessions of *Arabidopsis thaliana* (**Figure 5A**). Plants were cultivated with a longer light period at higher temperatures and lower photon irradiance than the initial conditions for Col-0 and C24. Phenotyping of the plants was conducted in the same manner through 7 days after salt treatment using RGB (**Figure 5A**) and ChlF imaging with the light curve protocol (**Supplementary Figure S6**). The rosette area of Te was the most significantly reduced by salt stress starting from day 3, while Col-0, Can, Co and Ler showed significant reductions later. Rosette areas of C24, Nd, and Cvi were not significantly reduced (**Figure 5B**). Natural variation was evident across all SIIT values in both intervals, indicating differences in salinity tolerance among the nine accessions. Lower SIIT_2_ than SIIT_1_ values indicated that plants became less tolerant over time; however, this difference was only significant for Cvi. This demonstrates that we were able to assess natural variation, not only in the growth reduction magnitude, but also in the timing of the responses to salt stress.

### Light Curve Chlorophyll Fluorescence Imaging Captured Early Responses to Salt Stress

**FIGURE 6:**
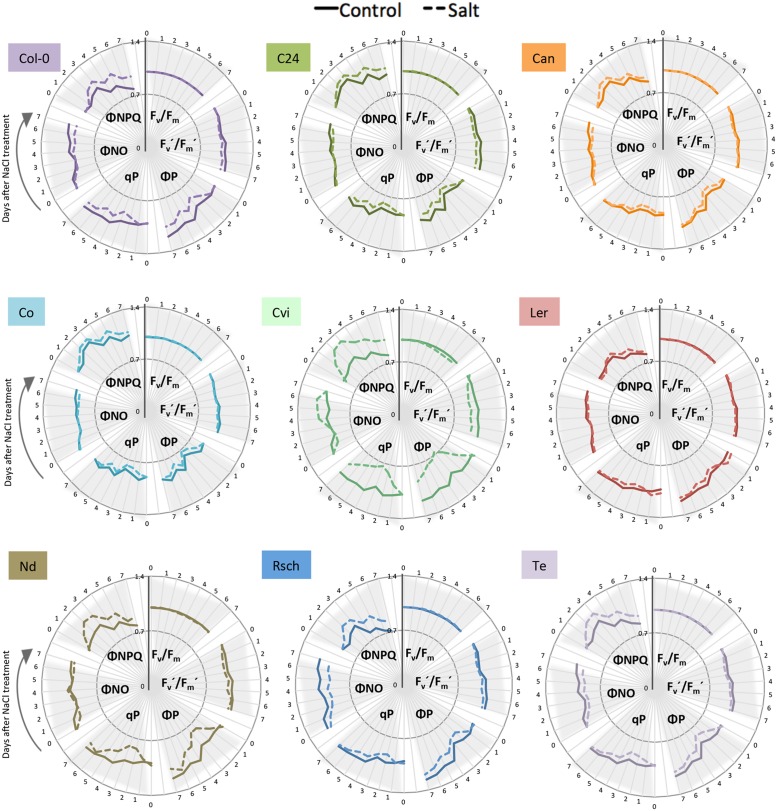
**Natural variation in ChlF-related responses of nine Arabidopsis accessions under salt stress.** Maximum quantum yield of PSII photochemistry for the dark-adapted (F_v_/F_m_) and light-adapted (F_v_′/F_m_′) states, the quantum yield of PSII photochemistry for the light-adapted state (ΦP), the photochemical quenching coefficient that estimates the fraction of open PSII reaction centers (qP), the quantum yield of constitutive non-regulatory non-photochemical dissipation processes (ΦNO) and the quantum yield of regulatory non-photochemical quenching (ΦNPQ) measured by the light curve protocol; control (solid line) and salt-stressed (dashed line) conditions. Time in days is indicated on the outer rim of the graph. Individual ChlF traits are presented in clusters indicated by gray triangles. Values represent the average of eight replicates per accession and treatment divided by the overall average per trait.

ChlF parameters were measured at four photon irradiances and were calculated as described previously (**Supplementary Table S2**). Differences in most ChlF parameters between control and salt-stressed plants were observed within 24 h of introducing salt at the highest photon irradiance (**Figure 6**). We found that the maximum quantum yield of photosynthesis in the dark-adapted state (F_v_/F_m_) was not affected by salt stress in Col-0. Rapid responses to salt stress were observed in photochemical and non-photochemical quenching, as ΦNPQ rapidly increased, followed by a decrease in non-regulatory heat dissipation ΦNO. The increase in heat dissipation via xanthophyll-mediated non-photochemical quenching (ΦNPQ) coincided with a significant decrease in the photochemical quenching coefficient (qP) and inhibition of the PSII operating efficiency (ΦP). Maximum quantum yield in the light-adapted state (Fv′/Fm′) decreased in response to salt stress, but not as severely as did the other ChlF traits (**Supplementary Figure S6B**; **Supplementary Table S4**).

Because the highest actinic photon irradiance (L4) provided the most discriminative power for the quantification of early salt-induced changes in ChlF parameters, it was chosen to assess the natural variation in photosynthetic activity. Comparison among the nine accessions using the six key ChlF traits revealed that F_v_/F_m_ did not differ between control and salt-treated plants, with the exception of Cvi (**Figure 6**; **Supplementary Table S4**). Increased ΦNPQ, upon exposure to salt stress, was observed to varying degrees (**Figure 6**; **Supplementary Table S4**). We also observed natural variation in the salt-induced decrease of ΦP, qP, and ΦNO over time. Overall, we observed that salt stress results in rapid and substantial increase in non-photochemical processes (i.e., the dissipation of heat in the PSII antennae), which correlates with reduced PSII quantum efficiency and photochemical quenching under stress (**Figure 6**; **Supplementary Figure S6C**).

**FIGURE 7:**
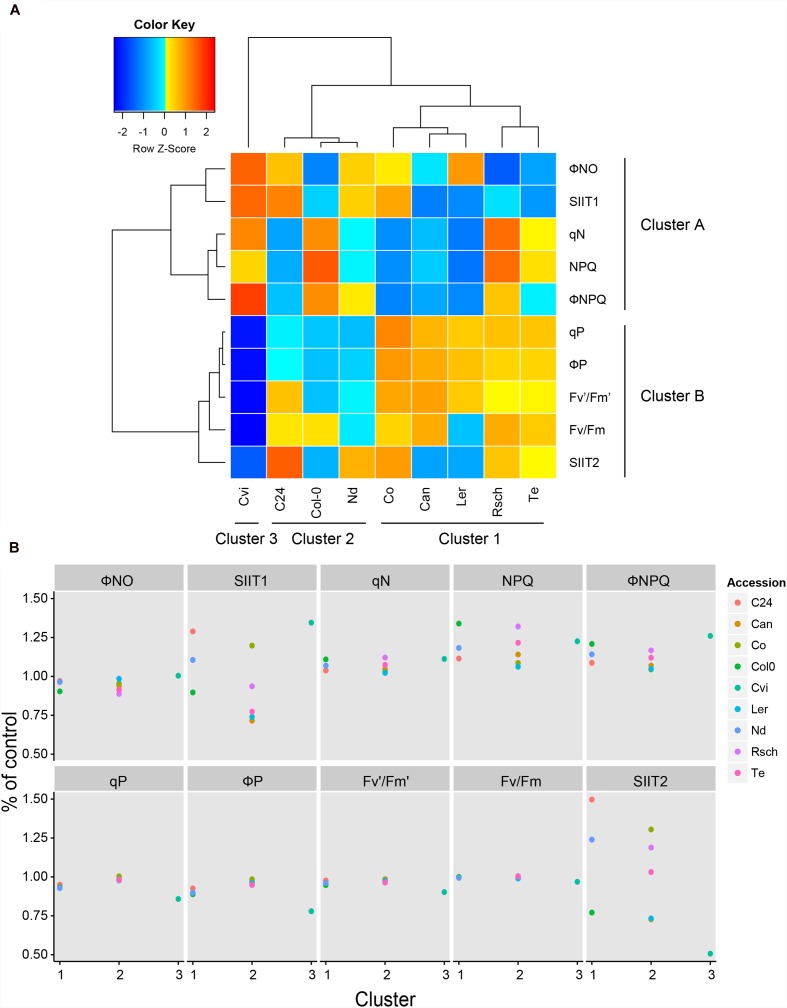
**Clustering of ChlF parameters with SIIT values revealed the early and late responses to salt stress.** **(A)** Using the Ward Linkage method, the shoot-ion independent tolerance (SIIT) values, SIIT_1_, SIIT_2_ and eight ChlF traits, measured at the highest photon irradiance (L4) at day 7, were clustered. Three cluster groups were identified for the nine accessions, while two clusters were observed to form across the phenotypic traits. The trait values for individual accessions presented in the heat map were normalized by the z-Fisher transformation per trait. **(B)** The effect of salt treatment on ChlF parameters and SIIT values across the three cluster groups identified in (A). SIIT_1_, SIIT_2_ and the selected ChlF parameters were calculated relative to control conditions and divided by the overall average per trait. Values represent average of eight biological replicates per accession and treatment.

Eight ChlF traits and two SIIT values were used to cluster the nine accessions using the Ward linkage method (**Figure 7A**). The accessions clustered into three groups (1–3) while the phenotypic traits were classified into two clusters (A and B). Clustering of traits revealed that non-photochemical quenching-related parameters (NPQ, ΦNO, ΦNPQ, and qN) are more prominent during the early stage (cluster A), while quantum yield-related parameters (F_v_/F_m_, F_v_′/F_m_′, ΦP, and qP) corresponded to the later stage of exposure to salt stress (cluster B). Eight accessions were grouped into two clusters (clusters 1 and 2) and distinct responses of Cvi placed it separately (cluster 3; **Figure 7**). Accessions in cluster 2 (C24, Nd, and Col-0) showed the least pronounced responses to salt stress during the early phase of exposure to salt stress in terms of SIIT_1_ (**Figure 5C**) and significant decline in photosynthetic activity (ΦP and qP; **Figures 6** and **7**; **Supplementary Table S4**). Finally, the accessions belonging to cluster 1 (Rsch, Te, Ler, Can, and Co) were characterized by rapid reduction in growth rate in the early phase of salt stress (**Figure 5C**) and less prominent changes in ChlF parameters (**Figures 6** and **7**; **Supplementary Table S4**). Hence, using ChlF parameters and SIIT values, we were able to distinguish between the processes affected in the early and late responses of plants to salt stress.

## Discussion

Recent advances in high-throughput phenotyping have allowed the parallel screening of multiple quantitative traits measuring plant growth and performance under stress conditions. In this study, we used RGB and ChlF measures, with rosette coloring, to dissect the complex responses of plants to salt stress. We developed a phenotyping protocol to monitor early physiological changes in response to salt stress involving growth, rosette morphology and photosynthetic performance. To make these evaluations, we determined the RGB, greenness and ChlF traits most responsive to salinity. The 60% soil-water content (100 mM NaCl) was the most suitable condition for studying early plant responses to salt stress without causing growth arrest (**Figure 2**) or premature leaf senescence (**Figure 3**).

Investigation of the RGB images revealed that salt stress caused little change in rosette morphology (**Figure 2B**; **Supplementary Table S1**); however, these phenotypes should not be overlooked as leaf slenderness and rosette compactness are known to play important roles in heat dissipation and transpiration rate (Bridge et al., 2013). Similarly, differences in rosette greenness were predominantly related to development and accession rather than to treatment with salt (**Figure 3**). Nevertheless, we did observe an increase in darker hues in plants grown in severe and moderate salt stress conditions than in those grown in mild stress, which could be due to accumulation of anthocyanin (Van Oosten et al., 2013). Pronounced changes in both lighter and darker hues were previously reported to occur in the later phases of salt stress response than the phenotyping period used in this study (Ben Abdallah et al., 2016). Therefore, color segmentation and quantification of green hues could provide valuable information regarding plant development and stress-related responses, especially when combined with quantitative pigment-content analysis.

We used automated image-processing pipelines to examine the salt-induced changes in rosette area over time by fitting a linear function to describe the growth over time (**Figures 2C** and **5B**; **Supplementary Table S1**). We used SIIT_1_ and SIIT_2_ as indicators of plant salinity tolerance in two time intervals (**Figures 2C** and **5B**) finding lower SIIT_2_ than SIIT_1_ values all accessions studied. The SIIT_2_ values of Cvi decreased dramatically, potentially due to its early increase in non-photochemical processes, which are represented by the traits NPQ, ΦNPQ and qN, with concurrent decreases in photochemical efficiency, represented by the traits F_v_′/F_m_′, ΦP, and qP. These steps are usually followed by increase in constitutive non-light-induced dissipation (ΦNO) and a drop in the maximal quantum efficiency of PSII in dark-adapted state (F_v_/F_m_; Mishra et al., 2012). The observed trend of lower SIIT_2_ values could be due to accumulation of ions in photosynthetic tissues, putting additional constraint on photosynthesis and subsequently plant growth (Munns and Tester, 2008).

Although, maximum quantum yield was commonly used for assessing plant performance under stress (Jansen et al., 2009; Bresson et al., 2015), we found that F_v_/F_m_ seems to be a robust parameter (**Figures 4** and **6**), being affected only under severe stress and not reflecting early salt stress responses, which is in agreement with previous reports (Baker and Rosenqvist, 2004). Other parameters quantifying photochemical and non-photochemical processes displayed more dynamic responses to salt stress (**Figures 4** and **6**). To identify the ChlF traits most responsive to salt stress, we performed PCA resulting in extracting PC1 and PC2, corresponding to the differences observed between treatment and accession, respectively (**Supplementary Figure S5**). According to PC1, F_o_, F_m_, F_v_, F_t,_ and F_p_ were the traits that changed the most in response to salt treatment. Based on traits contained in PC2, we found that the differences observed in F_v_/F_m_, NPQ, qN, and qP were more accession-specific (**Supplementary Table S3**). Based on our results, early ΦNPQ, qP, and ΦP responses to salt stress could be used to distinguish salt-tolerant from salt-sensitive plants (**Figures 4** and **6**). Similar results have been found for studies of drought and salt stress (Baker and Rosenqvist, 2004; Stepien and Johnson, 2009; Mishra et al., 2012). In conclusion, we observed largely different ChlF responses to salt stress among the accessions, evidencing that different accessions use different strategies to tolerate salt stress.

In this study, we demonstrated that phenotyping multiple quantitative traits in one experimental setup can provide new insights into the dynamics of plant responses to stress. These traits can be used to assess plant natural variation and to cluster accessions based on the magnitude and timing of their response to stress (**Figure 7**). Our work identified a set of phenotypes that can serve as markers for early responses to salt stress. These phenotypic markers can be used to study mutant populations, natural diversity panels and responses to other stress conditions, such as drought, cold or nutrient-deficiency, which would reveal the scope of their influence on tolerance to stress. Integrating thermal imaging into the phenotyping pipeline, along with quantifying water-use and transpiration-use efficiency, would provide a more comprehensive understanding of plant responses and development under stress. The protocol presented here can also be used to study non-model plants and crop species with more complicated 3D morphology, ultimately capturing a broad range of phenotypic traits. These traits could then be used in combination with forward genetics studies to identify genes underlying early responses to salt stress with the goal of providing new target genes for crop improvement.

## Author Contributions

MA performed the optimization protocol at PSI (Czech Republic) and most of the data analyses. MA, MJ, KP, and AN wrote the manuscript. With support from DS, AN performed the natural variation experiment at PSI. MA, MJ, and JF performed the phenotypic and statistical analyses. KP selected the nine accessions of *Arabidopsis thaliana* and designed the natural variation experiment. MTe, SN, and SS contributed to the original concept of the project and supervised the study. MTe, MTr, and KP conceived of the project and its components. All authors discussed the results and contributed to the manuscript.

## Conflict of Interest Statement

MTr is the owner and CEO of PSI (Photon Systems Instruments), Drasov, Czech Republic, and Dr. KP and JF are employees of his company. The other authors declare that the research was conducted in the absence of any commercial or financial relationships that could be construed as a potential conflict of interest.
