# Supplementary material for: High-Throughput Non-destructive Phenotyping of Traits that Contribute to Salinity Tolerance in Arabidopsis thaliana
Source: Front Plant Sci. 2016 Sep 28;7:1414. doi: 10.3389/fpls.2016.01414 (PMC5039194; doi:10.3389/fpls.2016.01414)
Supplement: Supplementary file 2 [file Presentation_1.PDF]

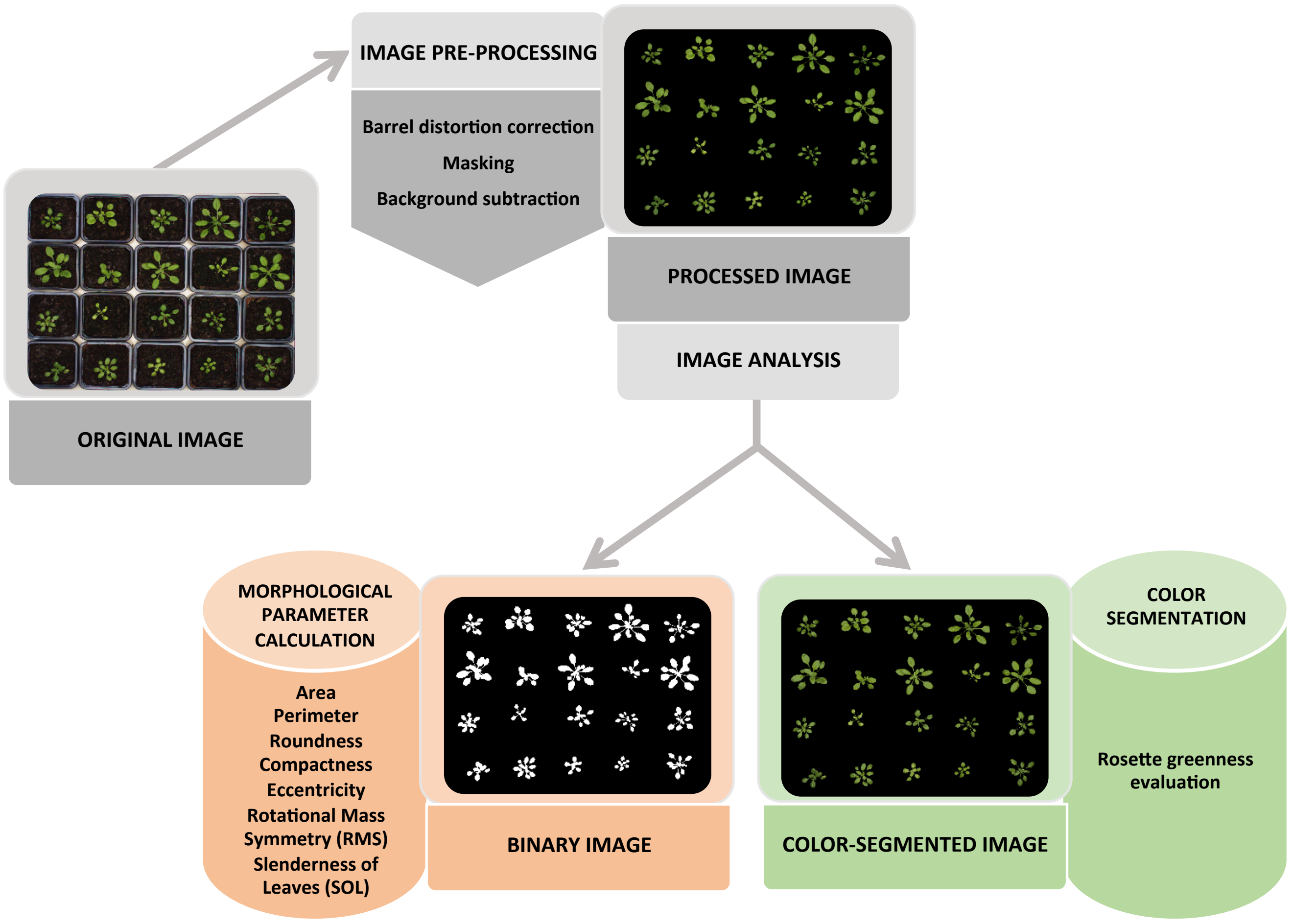

FIGURE S1 | Schematic of RGB image processing. Original RGB images were automatically processed using the PlantScreen^{TM} analyzer software to correct for barrel distortion caused by the fisheye lens, subtract the background and crop to isolate the plants within the tray, producing a binary (black and white) image. The binary images represent the plant surface (white) and background (black). Morphological analysis was conducted after separating the background from the plant shoot tissue. To evaluate greenness of the rosettes, RGB images were color-segmented to extract the green hues.

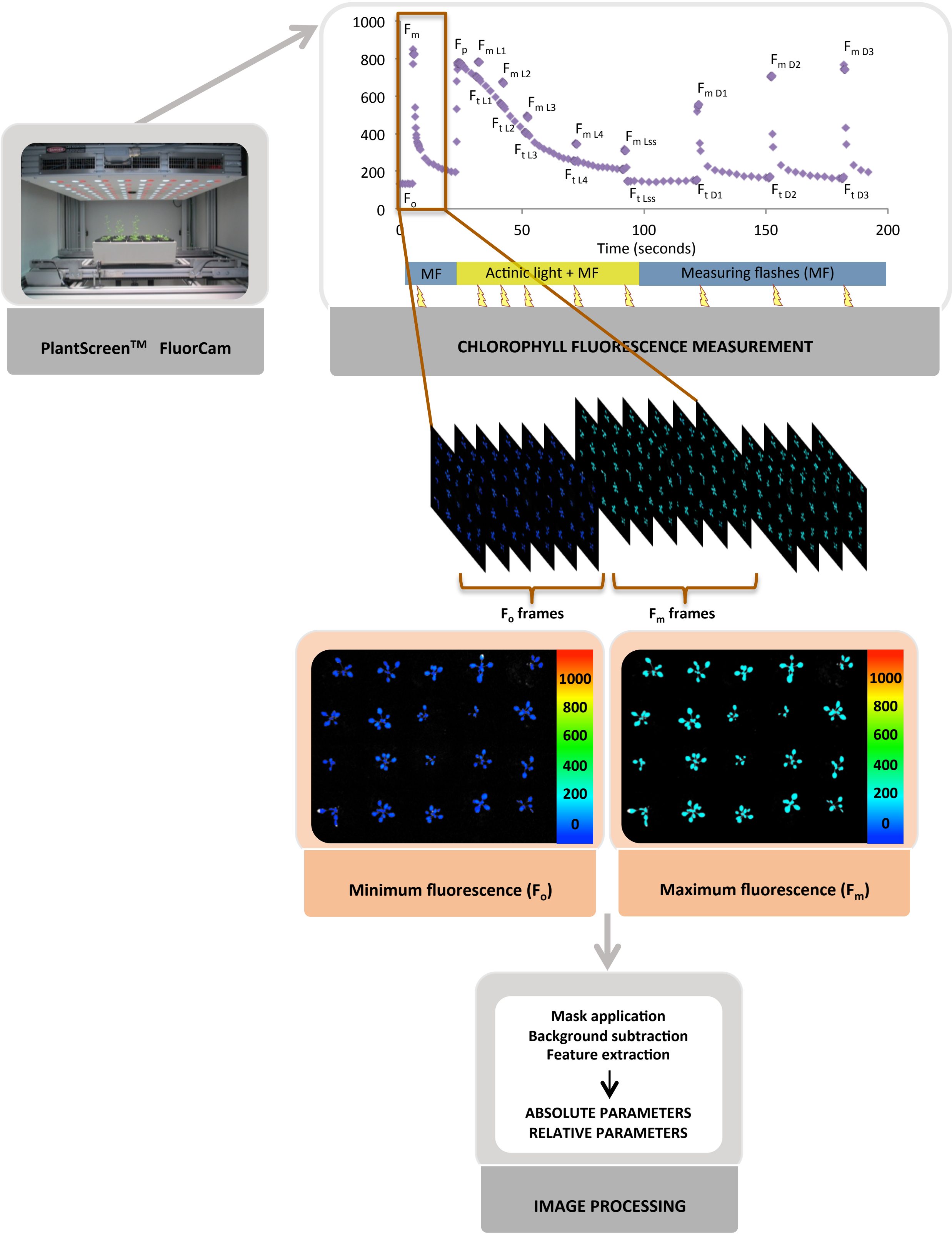

FIGURE S2 | Schematic of the kinetic ChlF protocol in the PlantScreen™ conveyer system. ChlF traits were captured with **a** PAM-based chlorophyll fluorometer. Images of the individual transient states were recorded. Corresponding frames were averaged for the measured parameters (F_o, F_m, F_m', $F_{t,}$ and F_p) or calculated from the captured frames to compute the relative parameters (F_o', F_v, F_v/F_m, Φ(P), F_v'/F_m', qP, Φ(NO), Φ(NPQ), NPQ, PQ, qN and Rfd). Automated ChlF image processing consisted of image segmentation by mask application, background subtraction and feature extraction. The signals from all pixels of each segment were averaged at each given time point. MF refers to the measuring flash, and yellow arrows indicate the saturation pulses that transiently saturated the electron transport chain.

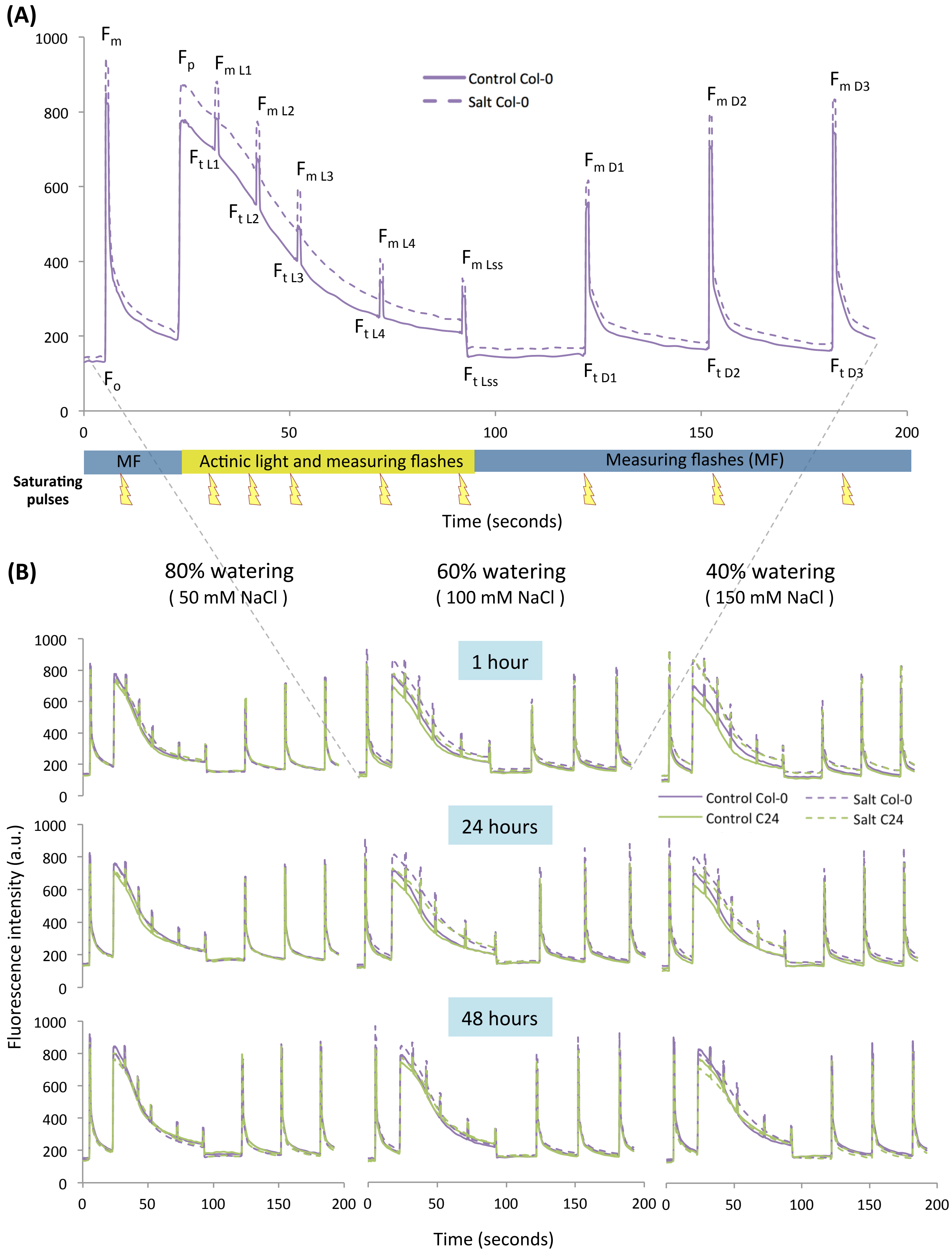

FIGURE S3 | ChlF parameters recorded early salt-induced changes in the photosynthetic performance. (A) Representative kinetic ChlF curve as recorded by quenching protocol. Induction kinetics for Col-0 at 60% watering with moderate salt stress (100 mM NaCl) for both control (solid lines) and salt stress (dashed lines) conditions are shown. Labels mark the ChlF levels measured either in the dark (F_o, F_m), during light adaptation (F_t_L, F_m_L) or during dark relaxation (F_t_D, F_m_D, F_o_D). Lss labels indicate the steady-state ChlF levels attained in continuous light (F_t_Lss, F_m_Lss, F_o_Lss). MF refers to the measuring flash. Yellow arrows indicate the timings of the saturation pulses that transiently saturated the electron transport chain. **(B)** Kinetic ChlF graphs of Col-0 (purple lines) and C24 (green lines) plants in both control (solid lines) and salt stress (dashed lines) conditions. The graphs represent ChlF transients recorded one, 24 and 48 hours after salt stress imposition. Values represent the average of nine replicates per accession and treatment.

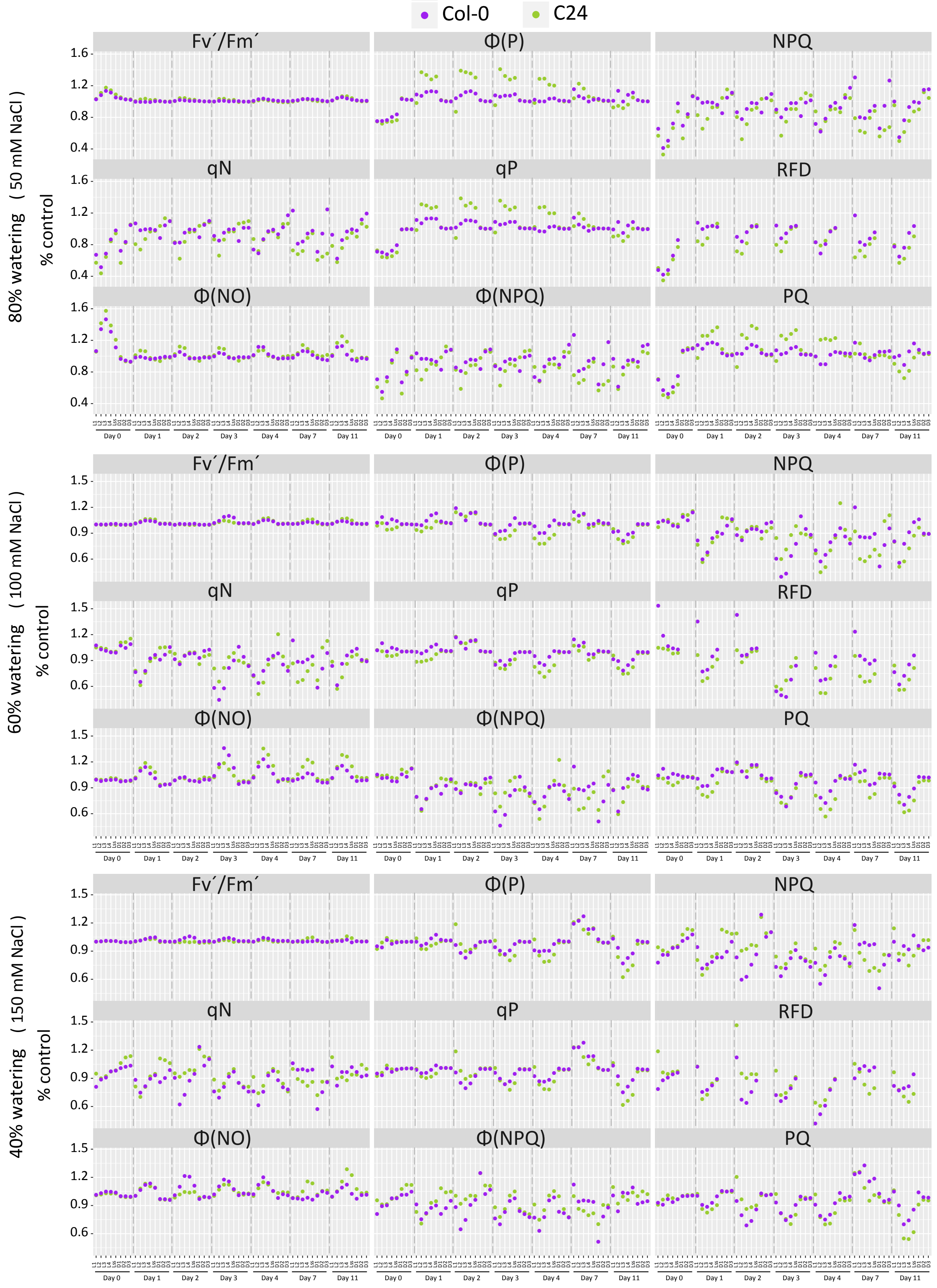

FIGURE S4 | Relative changes in ChlF parameters captured in light, dark and steady-states. Salt-induced changes in the maximal quantum yield of PSII photochemistry for the light-adapted state ($F_v{'}/F_m{'}$), the non-photochemical quenching estimating the rate constant for heat loss from PSII (NPQ), the quantum yield of PSII photochemistry for the light-adapted state (ΦP), the quantum yield of regulatory non-photochemical quenching (Φ(NPQ)), the non-photochemical quenching estimating fraction of variable chlorophyll fluorescence quenched by non-photochemical process (qN), the photochemical quenching coefficient, estimating the fraction of open PSII reaction centers (qP), the ratio of fluorescence decay (Rfd), the quantum yield of constitutive non-regulatory non-photochemical dissipation processes (Φ(NO)), the quantum yield of PSII photochemistry for the light-adapted state (ΦP), the quantum yield of regulatory non-photochemical quenching (Φ(NPQ)) and the photochemical quenching coefficient (PQ) were examined in Col-0 (purple) and C24 (green) grown under mild, moderate and severe salt stress conditions (50-, 100- and 150-mM NaCl, respectively). Values represent average of nine replicates per accession per condition, calculated as relative to control conditions, and divided by the overall average per trait.

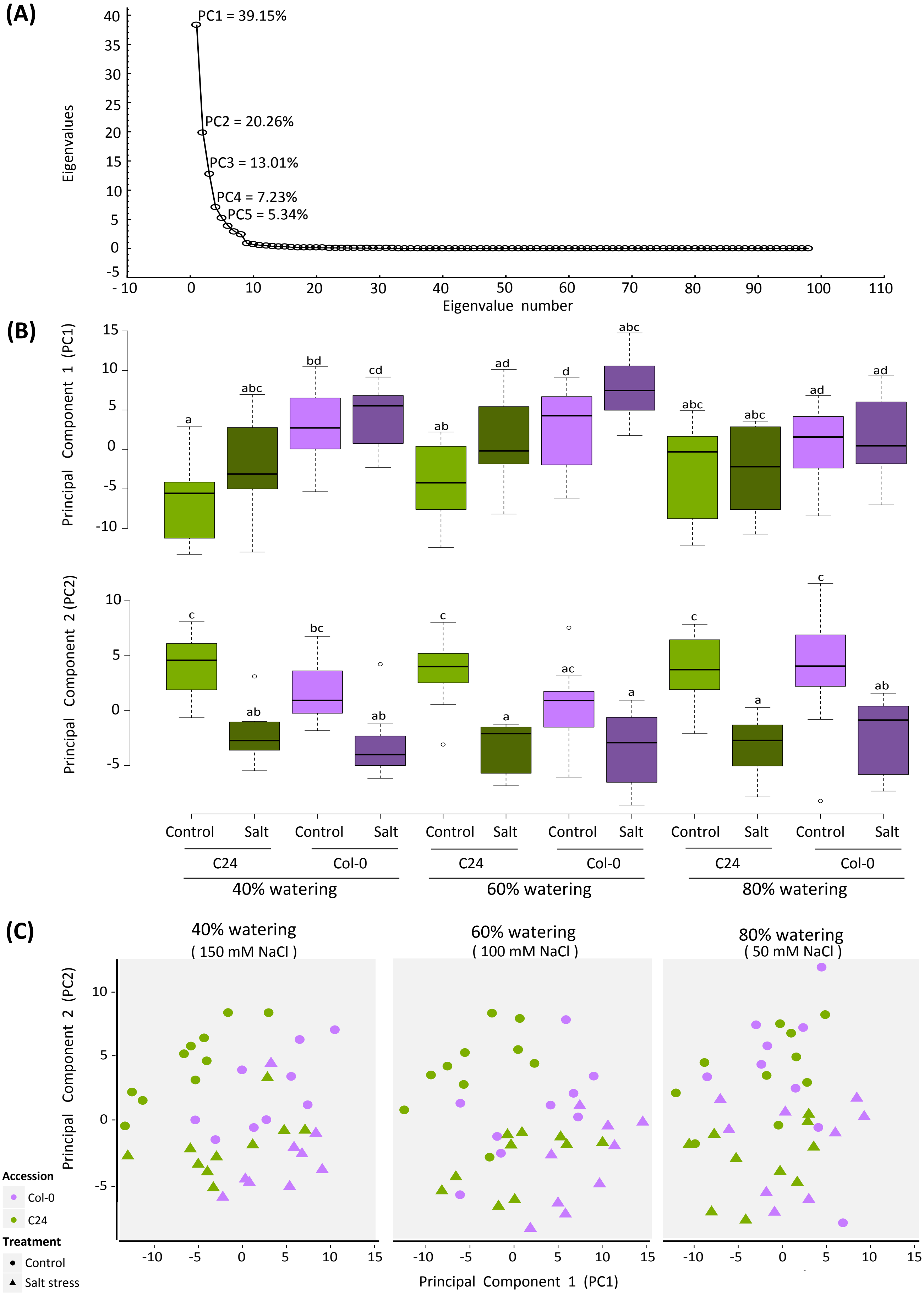

FIGURE S5 | Principal components of chlorophyll fluorescence (ChlF) traits show differences between accessions and salt stress conditions. (A) Eigenvalues of PCA for the increasing number of PCs. **(B)** Values of the first two PCs for Col-0 (purple) and C24 (green) across the three watering regimes and salt treatments of nine replicates. The letters indicate significantly different groups ($p < 0.05$). **(C)** Correlations between PC1 and PC2, with accessions indicated by color and conditions indicated by shape, across the three watering regimes.

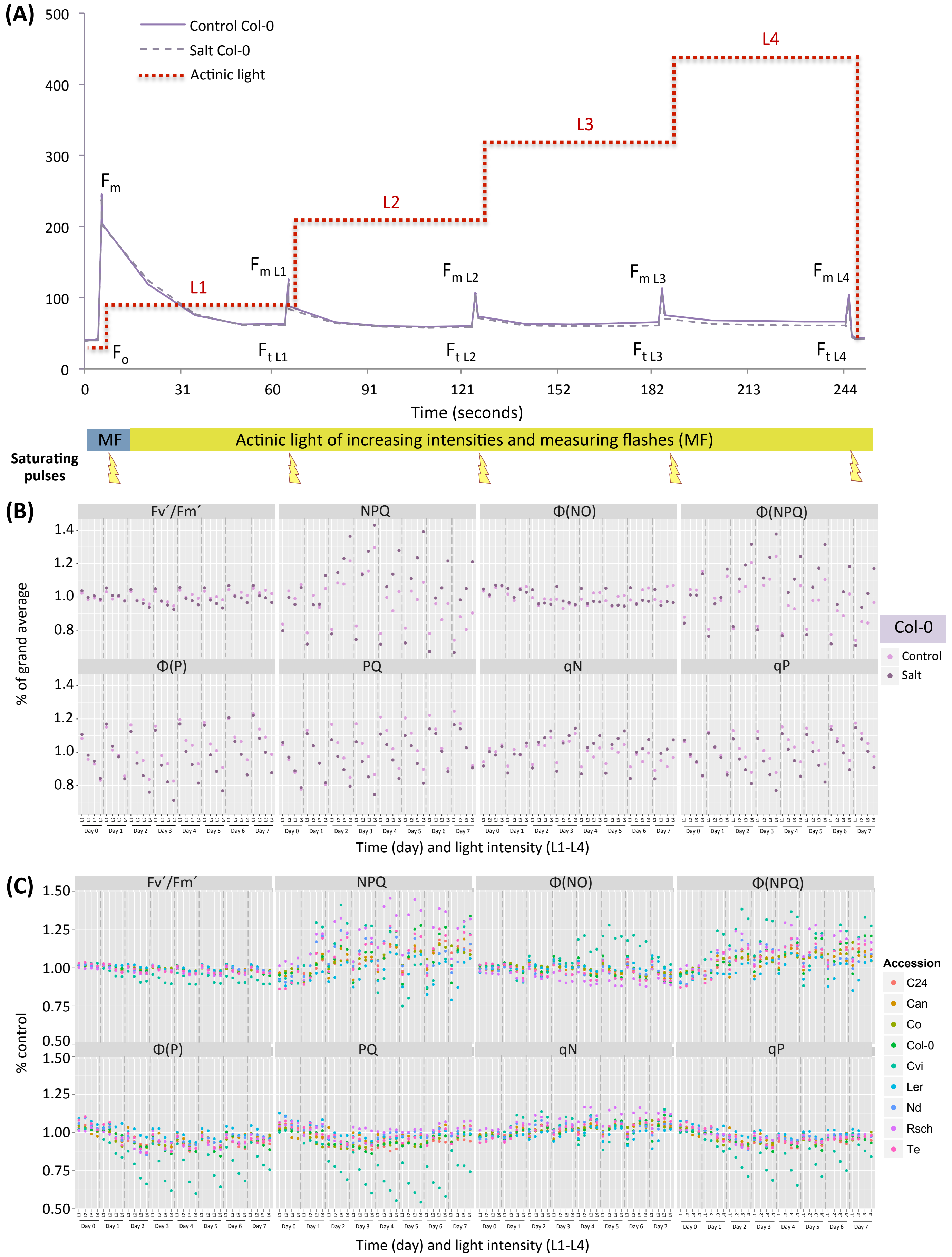

FIGURE S6 | Image-based analysis of ChlF parameters by the rapid light curve protocol. (A) Representative kinetic ChlF curve as recorded by using the light curve protocol in PAM mode. Induction kinetics, observed in Col-0 at 60% watering in control (solid lines) and salt stress (dashed lines) conditions, are presented. The photosynthetic performance was measured in the dark (F_o, F_m) as well as in continuous light with increasing light intensity from L1 to L4. Steady-state ChlF levels attained during the four light steps are shown in F_t and F_m Lss. MF refers to the measuring flash. Yellow arrows indicate the timings of the saturation pulses that transiently saturated the electron transport chain. ChlF transients were recorded after a

15-minutes dark-adaptation period, followed by 4 x (60 seconds) exposures to actinic light of increasing light intensities of L1, L2, L3 and L4 corresponding to 95, 210, 320, 440 µmol m^{-2} s^{-1}. **(B)** Salt-induced changes in the maximal quantum yield of PSII photochemistry for the light-adapted state (F_v'/F_m'), the non-photochemical quenching estimating the rate constant for heat loss from PSII (NPQ), the quantum yield of PSII photochemistry for the light-adapted state (ΦP), the quantum yield of regulatory non-photochemical quenching (Φ(NPQ)), the non-photochemical quenching estimating fraction of variable chlorophyll fluorescence quenched by non-photochemical process (qN), the photochemical quenching coefficient, estimating the fraction of open PSII reaction centers (qP), the quantum yield of constitutive non-regulatory non-photochemical dissipation processes (Φ(NO)) and the photochemical quenching coefficient (PQ) were recorded for Col-0 plants in control (light purple) and salt stress (dark purple). Values represent the average of eight biological replicates divided by the overall average per trait. **(C)** Salt stress-induced changes in the eight ChlF parameters among the nine accessions were determined by examining the trait value relative to control conditions per accession and normalizing by the overall average per trait. Values represent eight biological replicates per accession and treatment.

TABLE S1 | Summary of the RGB and greenness traits captured using the high-throughput phenotyping system. Mean trait values of Col-0 and C24 plants 11 d after control or salt treatments of nine replicates per accession and condition with standard error. Growth rates were determined using a linear function across two time intervals. Green hues, obtained by color segmentation, are represented as RGB coordinates.

TABLE S2 | Summary of the ChlF parameters captured by the high-throughput phenotyping system. Mean trait values of Col-0 and C24 plants 11 d after salt treatment of nine replicates per accession and condition with standard error.

TABLE S3 | Individual contributions of each trait per PC. On day 7, the PCs from Col-0 and C24 ChlF data were determined in response to salt stress. ChlF traits (F_o, F_m, F_v, F_p, F_t, F_q, F_v/F_m, F_v'/F_m', Φ(P), NPQ, qN, qP, and Rfd) in steady-states levels (Lss) and three dark-adapted states (D1, D2 and D3) were measured. The first five PCs explained 85% of the data variability.

TABLE S4 | Mann-Whitney U test of ChlF parameters with a continuity correction calculated for nine accessions. ChlF parameters, captured with the light curve protocol, were examined over 7 d of salt stress exposure. Presented *p*-values < 0.05 are highlighted in yellow, *p*-values < 0.01 are highlighted in orange and *p*-values < 0.001 are highlighted in red.
